# Trends in Decline of Antiretroviral Resistance among ARV-Experienced Patients in the HIV Outpatient Study: 1999–2008

**DOI:** 10.1155/2012/230290

**Published:** 2012-03-14

**Authors:** Kate Buchacz, Rose Baker, Douglas J. Ward, Frank J. Palella, Joan S. Chmiel, Benjamin Young, Bienvenido G. Yangco, Richard M. Novak, John T. Brooks

**Affiliations:** ^1^Divisions of HIV/AIDS Prevention, Centers for Disease Control and Prevention, 1600 Clifton Road, Mail Stop E45, Atlanta, GA 30333, USA; ^2^Division of Research, Cerner Corporation, Vienna, VA 22182, USA; ^3^Dupont Circle Physicians Group, Washington, DC 20009, USA; ^4^Feinberg School of Medicine, Northwestern University, Chicago, IL 60611, USA; ^5^DIDC, Rocky Mountain CARES, Denver, CO 80220, USA; ^6^Health Connections International, 1013 CP, Amsterdam, The Netherlands; ^7^Infectious Disease Research Institute, Tampa, FL 33614, USA; ^8^University of Illinois College of Medicine, Chicago, IL 60612, USA

## Abstract

*Background*. Little is known about temporal trends in frequencies of clinically relevant ARV resistance mutations in HIV strains from U.S. patients undergoing genotypic testing (GT) in routine HIV care. *Methods*. We analyzed cumulative frequency of HIV resistance among patients in the HIV Outpatient Study (HOPS) who, during 1999–2008 and while prescribed antiretrovirals, underwent GT with plasma HIV RNA >1,000 copies/mL. Exposure ≥4 months to each of three major antiretroviral classes (NRTI, NNRTI and PI) was defined as triple-class exposure (TCE). *Results*. 906 patients contributed 1,570 GT results. The annual frequency of any major resistance mutations decreased during 1999–2008 (88% to 79%, *P* = 0.05). Resistance to PIs decreased among PI-exposed patients (71% to 46%, *P* = 0.010) as exposure to ritonavir-boosted PIs increased (6% to 81%, *P* < 0.001). Non-significant declines were observed in resistance to NRTIs among NRTI-exposed (82% to 67%), and triple-class-resistance among TCE patients (66% to 41%), but not to NNRTIs among NNRTI-exposed. *Conclusions.* HIV resistance was common but declined in HIV isolates from subgroups of ARV-experienced HOPS patients during 1999–2008. Resistance to PIs among PI-exposed patients decreased, possibly due to increased representation of patients whose only PI exposures were to boosted PIs.

## 1. Introduction

Highly active combination antiretroviral therapy (cART) has significantly improved survival and reduced the rates of AIDS-related complications among HIV-infected persons [1–3]. Emergence of HIV variants with reduced susceptibility to antiretroviral (ARV) medications can significantly limit the effectiveness and durability of treatment [3–8]. Use of ARV resistance testing to optimize cART selection has been associated with better virologic and clinical outcomes [9–11] and improved survival [12], and resistance testing is now generally recommended in the clinical management of HIV infection [13–15]. We have previously shown that use of genotypic and phenotypic testing increased in the HIV Outpatient Study (HOPS) during 1999–2006 and that the likelihood of testing varied by HIV disease severity and demographic characteristics [16].

Recent European and Canadian studies have suggested that both the prevalence [17, 18] and incidence [19, 20] of ARV resistance among HIV-infected persons have declined, due predominately to a decrease in the proportion of patients with pre-cART mono- or dual-ARV experience, and the increasing use and effectiveness of more tolerable and potent cART regimens that appear less likely to result in resistance mutations [18–22]. However, relatively few recent data on prevalence or incidence of ARV resistance mutations are available from US patient populations [7, 23–25]. We sought to assess frequencies and trends in ARV resistance among HIV strains isolated from a demographically diverse cohort of ARV-experienced US patients who underwent genotypic testing (GT) as part of routine care between 1999 and 2008.

## 2. Methods

### 2.1. The HIV Outpatient Study

The HIV Outpatient Study (HOPS) is an ongoing, longitudinal cohort study of HIV-infected patients in care followed at HIV specialty clinics in the US since 1993. The HOPS methodology has been described previously [26]. In brief, trained staff abstract patient data, including sociodemographic characteristics, diagnoses, treatments, and laboratory values (including results of antiretroviral resistance testing), from medical records and enter them into an electronic database for central processing and analysis. HIV drug resistance testing is performed at the discretion of the clinician provider. The institutional research review boards of the Centers for Disease Control and the local participating sites have approved and reviewed the ethical conduct of this study yearly.

### 2.2. Study Population

For this analysis, we examined data from active participants seen at 10 HOPS clinics between January 1, 1999, and December 31, 2008, using the HOPS dataset as of September 30, 2009. Participants were considered active if they had at least two HOPS-related encounters documented (i.e., clinic visits, hospitalizations, and laboratory measurements but not telephone calls), and at least one of these encounters occurred during the calendar years of interest. The study population was restricted to patients who underwent GT while receiving ARV therapy with a documented plasma HIV RNA viral load >1,000 copies/mL; we chose the measurement most proximal to the date of GT within the six months prior and two weeks after the GT during 1999–2008. Only GT results documented on or after the first HOPS visit were considered; GT results indicating insufficient virus or inadequate specimen were excluded from the analyses.

### 2.3. Variable Definitions

 “Mono-dual NRTI exposure” was defined as any prior treatment with a regimen containing only one or two NRTIs as of the last eligible genotypic test in the year of interest. “Triple-class exposure” (TCE) was defined as four continuous months of exposure to ARVs from each of the three main classes (i.e., NRTIs, NNRTIs, and PIs) as of the last eligible genotypic test in the year of interest; resistance to other novel drug classes was not evaluated. “Only cART exposure” was defined as having never been prescribed non-cART (i.e., mono-dual NRTI) regimens as of the last eligible genotypic test in the year of interest. “Triple-class resistance” (TCR) was defined as the presence or presumed accumulation (i.e., any prior documentation) of mutations to ARVs from each of the three main classes.

### 2.4. Analysis Methods

Using the International AIDS Society (IAS) December 2008 listing of clinically relevant mutations, we assessed the frequency of major HIV resistance mutations in viruses isolated from patients who were followed in HOPS during 1999–2008 and underwent at least one eligible genotype test during that period. For patients who had multiple genotypic tests per year, we focused on the last test. Annual frequencies of ARV resistance were calculated as the number of patients with genotype(s) that during a calendar year demonstrated resistance, or had previously demonstrated resistance (numerator), divided by the number of patients with an eligible genotype performed in that year (denominator) and expressed as a percentage. Thus, resistance was determined by the presence of either a current or previously detected mutation(s) among patients tested that year, even if a previously detected mutation(s) was no longer apparent on the most recent resistance assay. We examined resistance to each of the three main drug classes (NRTIs, NNRTIs, and PIs) among all ARV-treated patients and among patients known to have been specifically exposed to agents in these drug classes. We also examined the frequency of TCR among TCE patients. Because M184V is a frequently observed mutation that develops rapidly after exposure to lamivudine and emtricitabine, but which does not confer resistance to all NRTIs, we conducted alternate analyses excluding this mutation. Finally, we assessed the likely impact of excluding genotypic tests performed at HIV RNA viral load ≤1,000 copies/mL on our findings, by examining frequency of insufficient specimens and resistance patterns in this subset.

Resistance was assessed as of the last eligible genotypic test in the year of interest. Trends in patient characteristics and in mutation frequency were evaluated using general linear models (PROC GLM), reporting *P* values over the 10-year period of the study. All analyses were performed using SAS version 9.1 (Cary, NC). Statistical associations with *P* values <0.05 were considered significant.

## 3. Results

### 3.1. Temporal Trends in Genotypic Resistance Testing

The 6,158 HOPS patients under observation during 1999–2008 had a total of 3,305 GT results during this period. We identified 1,867 (56%) genotypic tests performed while the patients were prescribed ARVs, of which 1,570 (84%) were performed when the most proximal HIV RNA was >1,000 copies/mL. These 1,570 GT results were obtained from a total of 906 patients, of whom 541 patients contributed one eligible GT result, 206 contributed two results, and 159 contributed ≥3 results. Taking the last GT result per patient per year resulted in 1,436 GT assessments for 906 unique patients being available for resistance trend analyses during 1999–2008.

**Figure 1:**
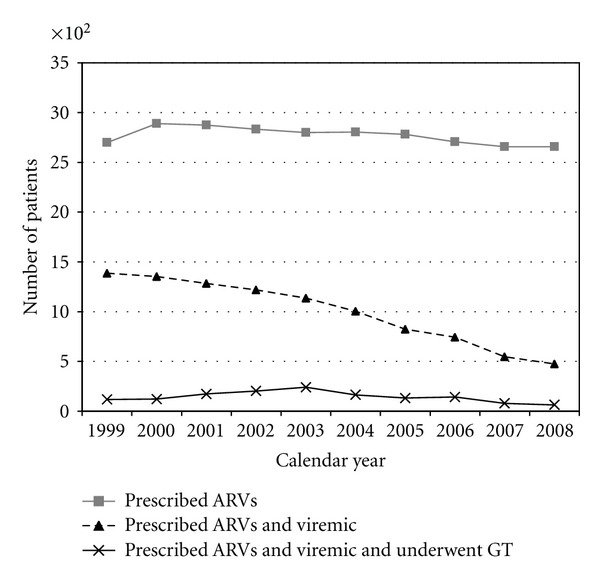
Number of patients who underwent genotypic testing (GT) while prescribed antiretrovirals (ARVs) and viremic (HIV viral load >1,000 copies/mL), the HIV Outpatient Study, 1999–2008.

Notably, while the annual number of active HOPS participants receiving ARVs in the HOPS oscillated around 2,700 during 1999–2008, the annual number and relative proportion of participants receiving ARVs who had at least one documented episode of viremia (HIV RNA >1,000 copies/mL) decreased markedly from nearly 1,400 to fewer than 500 participants (*P* < 0.0001, Figure 1). Correspondingly, since 2003, the number of persons who were eligible for GT and met inclusion criteria for our analyses decreased steadily (from 240 persons in 2003 to 63 persons in 2008, *P* < 0.0001). Furthermore, the percentage of genotypic tests performed among ARV-treated patients whose HIV RNA was ≤1,000 copies/mL (i.e., genotypic tests that we excluded from the present analyses) increased over time (4.5% in 1999 (6/133) versus 18.2% (16/88) in 2008, *P* = 0.008). See Section 3.5 for supplemental results for excluded patients with HIV RNA ≤1,000 copies/mL.

### 3.2. Characteristics of Patients with Genotypic Tests Performed

Of the 906 patients analyzed, the median age was 43 years, 77% were male, 37% were non-Hispanic black, 14% were Hispanic or other or unknown race/ethnicity, 54% were men who had sex with men (MSM), 11% were injection drug users at the time of HIV diagnosis, and 29% were heterosexuals (Table 1). As of their last eligible genotypic test, 49% had public insurance, 38% had a CD4+ cell count <200 cells/mm^3^, 80% had been diagnosed with AIDS by immunologic or clinical criteria, and the median HIV RNA was 4.3 log_10_ copies/mL at last eligible genotypic test. In crude analyses, the patients who underwent GT during 1999–2008 were increasingly more likely over time (test for trend, *P* < 0.05) to be older, female, of non-Hispanic black race/ethnicity, to have reported heterosexual risk for HIV infection, and to have been publicly insured (Table 1). The median CD4+ cell count appeared higher, and the HIV viral load appeared lower among patients who underwent GT in the middle of the study period (i.e., year 2004) compared with the start and the end of the period, without statistically significant trends over time (Table 1).

### 3.3. Temporal Trends in ARV Exposures

**Figure 2:**
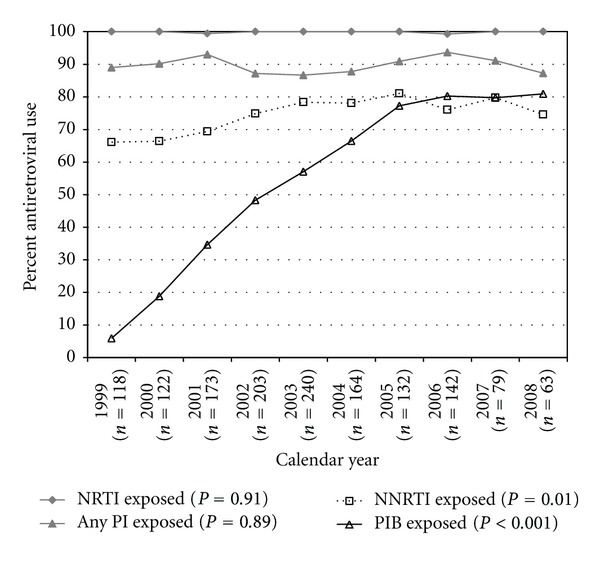
Frequency of antiretroviral exposures, according to ARV class, among viremic (HIV viral load >1,000 copies/mL) patients who underwent genotypic testing in the HIV Outpatient Study, 1999–2008. Note: Any “PI exposed” included ritonavir-boosted and unboosted regimens. “PIB exposed” included only ritonavir-boosted regimens.

**Figure 3:**
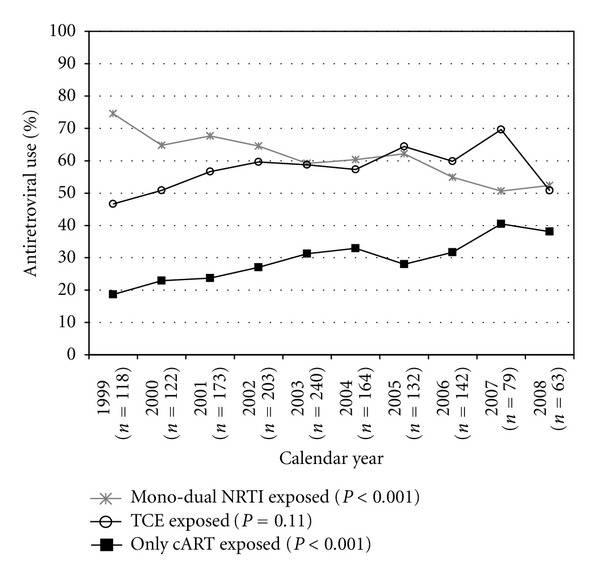
Frequency of ART experience categories, among viremic (HIV viral load* >*1,000 copies/mL) patients who underwent genotypic testing in the HIV Outpatient Study, 1999–2008.

From 1999 to 2008, among patients who underwent GT each year, the annual percentage of patients with prior NNRTI exposure increased (66% to 75%, test for linear trend, *P* = 0.012), while the percentage with any prior PI exposure remained stable (89% to 87%, *P* = 0.89) and the percentage with ritonavir-boosted PI exposure increased markedly (6% to 81%, *P* < 0.001) (Figure 2). The annual percentages with mono-dual NRTI exposure decreased (75% to 52%, *P* < 0.001), while the fraction of cART-only exposed participants increased (19% to 38%, *P* < 0.001), and the fraction of participants with TCE remained unchanged (47% to 51%, *P* = 0.11) (Figure 3).

### 3.4. Temporal Trends in Frequency of Genotypic Resistance

During 1999 to 2008, the annual frequency of any resistance mutations decreased for all patients tested (88% to 79%, test for linear trend *P* = 0.054) and for persons with mono-dual NRTI exposure (93% to 88%, *P* = 0.066) but not among persons who were exposed only to cART (68% to 63%, *P* = 0.32) or who had TCE (95% versus 94%, *P* = 0.43) (Table 2, part A). Although the differences were not statically significant, as evidenced by overlapping 95% CIs, the annual frequencies of resistance tended to be lower, among cART-only exposed patients compared with mono- and dual-NRTI-exposed patients or patients with TCE (Table 2, part A).

Among all patients receiving genotypic tests, regardless of their ARV regimen exposures, the frequency of any major resistance mutations (88% to 79%) (Table 2, part A top line) as well as NRTI mutations (82% to 67%) and PI mutations (65% to 41%) tended to decrease over time, but only the decline in PI resistance was statistically significant (Table 2, part B). The resistance findings were observed in the context of an increasing duration of cumulative months of exposure to all major classes of ARVs among the patients we studied (Table 1).

**Figure 4:**
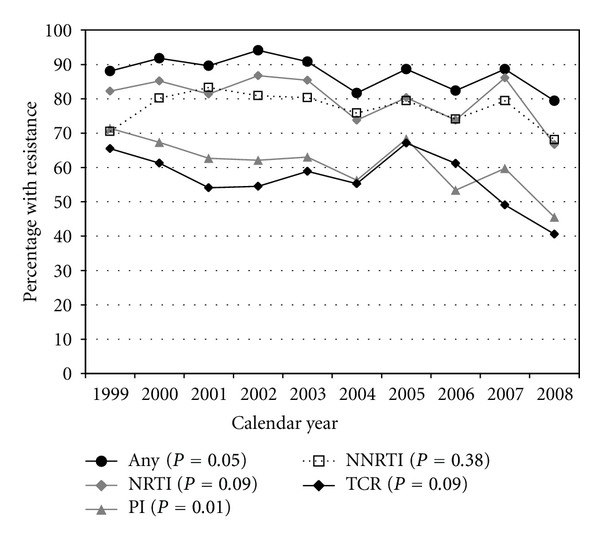
Frequency of genotypic resistance by class of antiretroviral, among exposed patients, in the HIV Outpatient Study, 1999–2008.

In analyses restricted to subsets of patients exposed to the relevant ARV drug classes, the frequencies of resistance to NRTIs (82% to 67%, test for linear trend *P* = 0.094) among NRTI-exposed patients and to NNRTIs (71% to 68%, *P* = 0.38) among NNRTI-exposed did not decrease significantly, but the frequency of resistance to PIs (71% to 46%, *P* = 0.010) among PI-exposed did (Table 2, part C; Figure 4). The annual frequency of TCR among patients with TCE also did not decrease significantly (66% to 41%, *P* = 0.094) (Figure 4).

In the sensitivity analyses that excluded the M184V mutation in reverse transcriptase, the frequency of NRTI resistance was 10–15% lower in each given calendar year, but the frequency of TCR decreased by <5% in each year (data not shown). Furthermore, when we restricted analyses to the first or the last genotypic test per patient during 1999–2008, to avoid correlated outcomes in the analyses, the declines in resistance observed in the primary analyses persisted.

The decrease in prevalence of PI resistance, both within the entire cohort and among persons exposed to PIs, correlated with a shift in exposures to specific PIs. The most common current or prior PI exposures in 1999 were to nelfinavir (70%), indinavir (69%), saquinavir (66%), and full-dose ritonavir (51%), whereas by 2008, the most common PI exposures were to low-dose ritonavir for PI boosting (75%), lopinavir (65%), atazanavir (53%), and indinavir (40%). Furthermore, we did not see a trend whereby exposure to PIs (as a class) was increasingly distanced from the date of genotype (i.e., differential risk for reversion to wild-type virus). In each calendar year, among patients who had any PI exposure to date, over 75% underwent GT while being prescribed at least one PI agent.

### 3.5. ARV Resistance according to HIV RNA Level

 In supplemental analyses, we compared the frequency (non-cumulative) of resistance mutations detected in each patient's most recent viral isolate from 2004–2008, according to HIV RNA level at GT, contrasting results for patients whom we included (HIV RNA >1,000 copies/mL) and excluded (HIV RNA ≤1,000 copies/mL) in our primary analyses. Of 624 unique patients who had their last GT while on ARVs in that period, 42 (7%), and 143 (23%) patients, respectively, had GT performed with the closest HIV RNA in the range of 500–1,000 copies/mL and <500 copies/mL. The percentages of patients missing GT results because of “insufficient specimen” were 2%, 12%, and 46% in samples with HIV RNA >1,000 copies/mL, 500–1,000 copies/mL, and <500 copies/mL, respectively. In the remaining evaluable isolates, the frequency of any major IAS resistance mutation was 76%, 76%, and 47% for the three HIV RNA strata, respectively.

## 4. Discussion

As treatment options for HIV-infected patients have improved over time in terms of greater effectiveness, tolerability, and sheer number of available antiretrovirals, we found that a decreasing proportion of ARV-treated patients in our diverse US-based cohort had viremia and thus were candidates for GT to optimize cART (Figure 1). Among ARV-experienced patients in our cohort who underwent GT in the context of routine outpatient HIV care during 1999–2008, viruses harboring resistance mutations to the main ARV classes were detected with either a constant or possibly decreasing frequency over time; resistance mutations to protease inhibitors decreased significantly in viral strains of patients who had ever been exposed to PIs.

The magnitude and temporal trends in the frequency of resistance mutations among HIV strains isolated from patients who undergo GT can be influenced by a myriad of factors, including but not necessarily limited to (i) changes in the sociodemographic and clinical characteristics of the patient population over time, particularly in a dynamic cohort such as the HOPS in which patients can enter or leave care; (ii) changes in the nature and extent of ARV exposure over time (e.g., decreasing exposure to mono- or dual-NRTI regimens, increasing exposure to newer more potent and tolerable cART regimens as first-line or salvage therapy); (iii) more widespread utilization of GT and changes in the characteristics of patients considered appropriate candidates for such testing; (iv) archiving of mutations in the absence of selective antiretroviral drug pressures; (v) cohort-level changes in the degree of patient adherence to prescribed ARVs and the related likelihood of experiencing viremia while prescribed cART.

It is important to note that our findings regarding the extent of and trends in the frequency of ARV resistance mutations apply only to the decreasing annual proportion of HOPS patients eligible for resistance testing [16], that is, persons who experienced viremia while receiving cART. Our aim was to describe what clinicians may see in practice when testing ARV-experienced patients. Modeling of population-wide prevalence of resistance in the HOPS cohort (including both viremic and virologically suppressed patients on cART), which was not undertaken here but has been attempted in other populations [25, 27], would presumably reveal lower prevalence of resistance cohort-wide and potentially different trends over time. Such modeling approaches typically require strong assumptions about the likelihood of archived resistance mutations among patients who are currently virologically suppressed and imputations of missing data for many ARV-experienced patients who did not undergo GT (the bulk of ARV-experienced patients in the HOPS, per Figure 1) but can provide useful comparative data in large samples under different assumed scenarios.

Although we did not detect statistically significant declines in the frequency of any ARV resistance among HIV strains from all HOPS patients who underwent GT in clinical practice and among the subset who were solely cART-experienced, recent population-based studies provide supporting evidence for such declines. These studies suggest that the incidence of ARV resistance is decreasing [19, 20] due to increasing use of cART regimens that are more tolerable, potent, easier to adhere to, and consequently less likely to result in resistance [18, 21, 22] and due to smaller proportions of patients failing antiretroviral therapy [7, 19] or being sufficiently viremic while receiving cART to allow for the performance of GT.

We also found that HOPS patients who underwent GT were increasingly less likely to have virus with PI resistance, whereas potential declines in NNRTI resistance were more modest and nonsignificant over time. These trends might reflect shorter persistence of PI mutations than NNRTI mutations in the absence of drug pressure or greater use of ritonavir-boosted PIs, which are more potent and confer greater barriers to the emergence of resistance. Among ARV-naïve patients initiating cART who were followed in British Columbia, Canada, the development of ARV drug resistance was associated with the use of non-boosted PI-based or NNRTI-based regimens [18], suboptimal levels of ARV adherence (particularly in the 80%–95% range), low pre-cART CD4+ cell counts, and high baseline HIV RNA levels [18, 28], and was reduced for patients who initiated cART in 2002–2004 versus earlier [18]. In the Swiss Cohort study, the estimated prevalence of resistance in a population of over 8,000 ARV-exposed patients also decreased by approximately 12% during 1999 to 2007 and was driven by loss to follow up or death of patients with mono-dual NRTI exposure countered by continued enrollment of patients starting cART with NNRTI-based or boosted PI-based regimens [17].

We excluded from our primary analyses genotypic tests performed on plasma HIV RNA samples with ≤1,000 copies/mL because such assays may provide unreliable results (i.e., appear falsely negative for resistance mutations) due to insufficient HIV RNA copy and such patients may have low level self-limited viremia (viral “blips”) rather than true virologic failure. Indeed, when comparing results from genotypic tests performed when HIV RNA >1,000 copies/mL versus ≤1,000 copies/mL in the latest years of the study, we found that the frequency of “insufficient specimens” was higher, while the frequency of detected resistance was lower in the latter group, especially when HIV RNA <500 copies/mL. Aside from concern for bias due to nonevaluable samples, including the relatively few results from genotypic tests performed with HIV RNA between 500 and 1,000 copies/mL would have likely accentuated the observed decreases in the frequencies of resistance at GT through 2008. Our analytic approach ensured more comparable data over time, and the declines in resistance we observed are likely conservative.

Our findings suggestive of declines over time in rates of ARV resistance are notable for at least two reasons. First, because of the method we employed to analyze cumulative frequency of ARV resistance to date, by “carrying forward” previously identified mutations that may have become archived, the frequency of resistance in later years should tend to be higher (because patients have had the opportunity to accumulate more mutations over time as revealed by successive genotypic tests performed) than would be suggested if results were based on a single genotypic test that year. Further, one might expect to find higher overall frequency of mutations over time, as newer ARVs are added to the three drug classes, with new, often ARV-specific, resistance mutations being identified and detected using contemporary genotypic tests.

We believe that three factors most likely explain the lower frequency of resistance among HOPS patients who underwent GT over time: (i) the increasing use of more tolerable and potent cART regimens associated with higher barriers to resistance or more complex mutational pathways necessary for resistance to develop; (ii) the decreasing proportion of HOPS patients under observation having mono-dual NRTI exposure; (iii) the increasing and more widespread use of GT in the HOPS with possible testing of patients at lower risk of virologic failure due to resistance. The decreasing frequency of resistance to PIs in our population was associated with increased use of more potent PIs (i.e., ritonavir boosting). This trend could have also occurred if exposures to PIs were increasingly distant, which would have allowed greater opportunity for PI resistance mutations to become archived before patients underwent GT. However, we found no evidence that the time between PI exposure among exposed patients and time of genotypic test differed over the study observation period.

In a cohort study of 1,587 HIV-infected patients at the University of North Carolina (2000–2006), of whom 607 had at least one genotype, there were 27.2% of patients with TCR among 437 patients who had GT and any exposure to each of the three drug classes. This was lower than the 40.6% estimated for patients with TCE in our study, possibly because we required a minimum of three months of exposure on each of the three drug classes to define TCE [25]. Our findings of high frequency of any resistance mutations in the genotyped population are consistent with the findings from an early large genotypic substudy of patients with HIV viral load >500 copies/mL in the population-based HIV Cost and Service Utilization Study (HCSUS), which documented that 76% of tested patients had evidence of genotypic resistance to one or more antiretroviral drugs in 1996–1998 [23].

The findings from our ecological analyses should be interpreted in light of several additional caveats and limitations. First, most patients receiving antiretrovirals in the HOPS were virologically suppressed and, over time, fewer were eligible for GT during the study period, resulting in a progressively smaller and more select sample of patients with GT results, raising the concern for potential bias and limiting statistical power. Second, we relied on data collected in the course of regular HIV care since our purpose was to reflect what a treating clinician would see. Not all eligible patients had GT performed, and trends in and magnitude of resistance frequencies detected may have been different if all ARV-experienced, viremic patients had undergone scheduled GT at regular intervals [16]. Third, some mutations that arose during prior courses of ARV therapy (before the advent of GT or before HOPS entry) may have no longer been apparent because of a lack of continuing ARV drug pressure; however, we do not believe this effect would have systematically biased our findings over calendar years away from the null hypothesis. Fourth, patients who harbored the greatest number of mutations were also more likely to have had advanced HIV disease and an extensive ARV treatment history that involved mono-dual NRTI exposure (data not shown). These patients may have died or been lost to follow up at rates higher than other HOPS patients during the study period. Moreover, the demographics of patients who underwent GT shifted toward a higher percentage of women, persons of color, and those with heterosexual risk for HIV infection, all factors which are associated with more recent HIV diagnoses and less exposure to ART in the HOPS (data not shown). These dynamics may explain in part the decreasing trends in resistance in our open cohort. Finally, we have no systematic data on pre-ARV resistance profiles for the majority of patients and therefore could not evaluate the role of primary transmitted drug resistance or incidence rates of HIV resistance.

In conclusion, the frequency of ARV resistance mutations detected for HIV among patients in the HOPS who were tested in the course of routine clinical practice was high and tended to decrease during 1999–2008. The decreasing frequency of genotypic resistance corresponded with at least two trends: an increasing use of more tolerable and potent cART, including boosted PI regimens, and a decreasing proportion of HOPS patients with histories of exposure to mono- and dual-NRTI ARV regimens. Our findings support the continuing need for routine HIV resistance testing and monitoring of evolving ARV resistance patterns among ARV-treated patients [15] who experience virologic nonsuppression while on therapy. Available evidence suggests that adoption of such testing as routine would facilitate tailoring of more effective cART [29], reduce the likelihood of further resistance evolution, and ultimately augment cART-related reductions in HIV-related mortality [7, 8, 12].

## Figures and Tables

**Table 1:** Characteristics of ARV-experienced patients who had genotypic testing performed at viral load >1000 copies/mL and while on ARVs in selected calendar years, and overall, the HIV Outpatient Study, 1999–2008.

	1999		2004		2008		Unique total^§^	
	*N*	%	*N*	%	*N*	%	*N*	%
Had GT in this yr →	118		164		63		906	
Age, years^∗†^								
Median (IQR)	40	(36, 47)	43	(39, 48)	41	(38, 51)	43	(38, 49)
<35	18	15.2	17	10.4	9	14.3	110	12.1
35–49	78	66.1	110	67.1	36	57.1	583	64.4
50+	22	18.6	37	22.6	18	28.6	213	23.5

Gender^†^								
Female	25	21.2	36	22.0	24	38.1	208	23.0
Male	93	78.8	128	78.0	39	61.9	698	77.0

Race^†^								
White	73	61.9	75	45.7	22	34.9	448	49.4
Black	30	25.4	65	39.6	30	47.6	334	36.9
Other/unknown	15	12.7	24	14.6	11	17.5	124	13.7

HIV Risk^†^								
Heterosexual	31	26.3	53	32.3	27	42.9	266	29.4
IDU	12	10.2	15	9.1	9	14.3	102	11.3
MSM	70	59.3	85	51.8	23	36.5	487	53.8
Other/unknown	5	4.2	11	6.7	4	6.4	51	5.6

Insurance^∗†^								
Private	54	45.8	58	35.4	21	33.3	381	42.0
Public	49	41.5	93	56.7	37	58.7	444	49.0
Other/unknown	15	12.7	13	7.9	5	7.9	81	8.9

CD4+ count, cells/mm^3∗^								
Median (IQR)	225.5	(105, 360)	272	(163, 432)	170	(64, 325)	267.5	(124, 433)
<200	54	45.8	56	34.2	36	57.1	347	38.3
200–349	32	27.1	46	28.0	11	17.5	229	25.3
350–499	16	13.6	34	20.7	7	11.1	162	17.9
500+	15	12.7	26	15.8	8	12.7	150	16.6
Missing	1	0.8	2	1.2	1	1.6	18	2.0

Nadir CD4+ count, cells/mm^3∗†^								
Median (IQR)	99	(34, 213)	126	(40, 247)	55	(20, 206)	105	(32, 247)
<50	38	32.2	52	31.1	29	46.0	278	30.7
50–199	44	37.3	52	31.7	18	28.6	306	33.8
200–349	24	20.3	38	23.2	12	19.0	184	20.3
350+	9	7.6	20	12.3	4	6.4	101	11.2
Missing	3	2.5	3	1.8	0	0.0	37	4.1

HIV RNA viral load, copies/mL*								
Median log (IQR)	4.6	(3.9, 5.0)	4.2	(3.6, 4.8)	4.5	(3.7, 5.0)	4.3	(3.6, 4.9)
1,000–9,999	35	29.7	65	39.6	23	36.5	368	40.6
10,000–99,999	54	45.8	69	42.1	23	36.5	363	40.1
≥100,000	29	24.6	30	18.3	17	27.0	175	19.3

AIDS*	99	83.9	133	81.1	52	82.5	728	80.4

Antiretroviral exposure*								
3TC/FTC exposed	110	93.2	158	96.3	62	98.4	856	94.5
NRTI exposed	118	100.0	164	100.0	63	100.0	905	99.9
NNRTI exposed	78	66.1	128	78.1	47	74.6	656	72.4
PI exposed (any)	105	89.0	144	87.8	55	87.3	799	88.2
Ritonavir-boosted PI exposed	7	5.9	109	66.5	51	81.0	478	52.8
Mono-dual NRTI exposed	88	74.6	99	60.4	33	52.4	515	56.8
Triple-class exposed (TCE)	55	46.6	94	57.3	32	50.8	496	54.8
Only cART exposed	22	18.6	54	32.9	24	38.1	299	33.0
Any exposure to all 3 classes of agents (NRTI, NNRTI, and PI)	71	60.2	109	66.5	39	61.9	566	62.5

ARV exposure duration among those exposed with complete and evaluable ARV history, median months*	*n*	Median	*n*	Median	*n*	Median	*n*	Median
3TC/FTC exposed	110	22	158	43	62	58	856	37
NRTI exposed	118	44	164	71	63	73	905	62
NNRTI exposed	78	12	128	19	47	23	656	20
PI exposed (any)	105	28	144	51	55	56	799	43
Ritonoavir-boosted PI exposed	7	5	108	23	51	36	477	21
Mono-dual NRTI exposed	88	19	98	12	31	7	503	13
Triple-class exposed (TCE)	38	11	75	46	29	57	394	38
Only cART exposed	11	15	54	36	24	39	299	34

Number of ARVs exposed to date, median	118	6	164	8	63	7	906	7

Duration of ARV exposure, years, median	118	4.0	164	6.2	63	6.2	906	5.3

^§^ Total number of unique patients during 1999–2008.

*As of last eligible genotypic test in the year. (For clinical measurements, only values within 6 months prior through 2 weeks after the first eligible genotypic test were considered, the closest one chosen to characterize the patient at time of the GT.).

^†^ *P* value for trend during 1999–2008 was <0.05.

IQR: interquartile range. GT: genotypic testing; cART: combination antiretroviral therapy; NRTI: nucleoside reverse transcriptase inhibitor; NNRTI: non-nucleoside reverse transcriptase inhibitor; PI: protease inhibitor; TCE: triple-class exposed; MSM: men who have sex with men; IDU: injection drug use.

**Table 2:** Frequency of resistance mutations among ARV-experienced patients who had genotypic testing performed at viral load >1,000 copies/mL and while on ARVs, by year, the HIV Outpatient Study, 1999–2008.

Patient subgroup	1999	2004	2008	*P* trend*
	% [*N*]	% [*N*]	% [*N*]	
Part A. Frequency of any resistance among all tested in the year—by patient subgroup				

All patients tested	88.1 [118]	81.7 [164]	79.4 [63]	0.054
95% CI	(80.9, 93.4)	(74.9, 87.3)	(67.3, 88.5)	
*By type of ARV experience:*				
cART only	68.2 [22]	75.9 [54]	62.5% [24]	0.317
95% CI	(45.1, 86.1)	(62.4, 86.5)	(40.6, 81.2)	
Mono-dual NRTI	93.2 [88]	86.9 [99]	87.9 [33]	0.066
95% CI	(85.8, 97.5)	(78.6, 92.8)	(71.8, 96.6)	
TCE	94.5 [55]	88.3 [94]	93.8 [32]	0.426
95% CI	(84.9, 98.9)	(80.0, 94.0)	(79.2, 99.2)	

Part B. Frequency of class-specific mutations—among all patients tested in the year				

NRTI resistance	82.2 [118]	73.8% [164]	66.7% [63]	0.096
95% CI	(74.1, 88.6)	(66.4, 80.3)	(53.7, 78.0)	
NNRTI resistance	46.6 [118]	65.2% [164]	55.6% [63]	0.165
95% CI	(37.4, 56.0)	(57.4, 72.5)	(42.5, 68.1)	
PI resistance	65.2 [118]	50.6% [164]	41.3% [63]	0.018
95% CI	(55.9, 73.8)	(42.7, 58.5)	(29.0, 54.4)	
Triple-class resistance	33.9 [118]	39.6% [164]	27.0% [63]	0.748
95% CI	(25.4, 43.2)	(32.1, 47.6)	(16.6, 39.6)	

Part C. Frequency of class-specific mutations—among tested with prior ARV exposures				

NRTI resistance	82.2 [118]	73.8% [164]	66.7% [63]	0.094
95% CI	(74.1, 88.6)	(66.4, 80.3)	(53.7, 78.0)	
NNRTI resistance	70.5 [78]	75.8% [128]	68.1% [47]	0.384
95% CI	(59.1, 80.3)	(67.4, 82.9)	(52.9, 80.9)	
PI resistance	71.4 [105]	56.3% [144]	45.5% [55]	0.010
95% CI	(61.8, 79.8)	(47.7, 64.5)	(32.0, 59.4)	
Triple-class resistance	65.5 [55]	55.3% [94]	40.6% [32]	0.094
95% CI	(51.4, 77.8)	(44.7, 65.6)	(23.7, 59.4)	

*Linear test for trend using annual estimates for entire period 1999–2008.

Numbers in square brackets indicate denominators (i.e., patients with eligible GT each year).

CI: confidence interval. GT: genotypic testing; cART: combination antiretroviral therapy; NRTI: nucleoside reverse transcriptase inhibitor; NNRTI: non-nucleoside reverse transcriptase inhibitor; PI: protease inhibitor; TCE: triple-class exposed.

## Appendix

### The HIV Outpatient Study (HOPS) Investigators, 2011

The HOPS Investigators currently include the following investigators and sites: John T. Brooks, Kate Buchacz, Marcus Durham, Division of HIV/AIDS Prevention, National Center for HIV, STD, and TB Prevention (NCHSTP), Centers for Disease Control and Prevention (CDC), Atlanta, GA; Kathleen C. Wood, Rose K. Baker, James T. Richardson, Darlene Hankerson, and Carl Armon, Cerner Corporation, Vienna, VA; Frank J. Palella, Joan S. Chmiel, Carolyn Studney, and Onyinye Enyia, Feinberg School of Medicine, Northwestern University, Chicago, IL; Kenneth A. Lichtenstein and Cheryl Stewart, National Jewish Medical and Research Center Denver, CO; John Hammer, Benjamin Young, Kenneth S. Greenberg, Barbara Widick, and Joslyn D. Axinn, Rose Medical Center, Denver, CO; Bienvenido G. Yangco and Kalliope Halkias, Infectious Disease Research Institute, Tampa, FL; Douglas J. Ward and Jay Miller, Dupont Circle Physicians Group, Washington, DC; Jack Fuhrer, Linda Ording-Bauer, Rita Kelly, and Jane Esteves, State University of New York (SUNY), Stony Brook, NY; Ellen M. Tedaldi, Ramona A. Christian, Faye Ruley and Dania Beadle, Temple University School of Medicine, Philadelphia, PA; Richard M. Novak and Andrea Wendrow, University of Illinois at Chicago, Chicago, IL.

## Abbreviations

NNRTI: non-nucleoside reverse transcriptase inhibitor
PI: protease inhibitor
PIB: ritonavir-boosted protease inhibitor
NRTI: nucleoside reverse transcriptase inhibitor
TCE: triple-class exposed.
